# Correction of aberrant splicing caused by intronic *CAPN3* pathogenic variants using RNA-targeted therapeutic strategies in limb-girdle muscular dystrophy type R1

**DOI:** 10.1186/s13023-026-04451-x

**Published:** 2026-06-13

**Authors:** Gaoyuan Li, Yunuo Guo, Guangyu Wang, Haoyang Liu, Xiaoqing Lv, Pengfei Lin

**Affiliations:** Department of Neurology, Research Institute of Neuromuscular and Neurodegenerative Diseases, Qilu Hospital of Shandong University, Shandong Provincial Key Laboratory of Mitochondrial Medicine and Rare Diseases, Jinan, Shandong 250012 China

**Keywords:** RNA splicing, Splice-switching oligonucleotides, Modified U1 snRNAs, LGMDR1

## Abstract

**Background:**

Pre-mRNA splicing is a highly precise process, and it is estimated that approximately 9%–11% of pathogenic variants in patients with rare genetic diseases are caused by non-coding variants that disrupt this mechanism. Developing targeted strategies to correct such splicing defects represents a promising therapeutic avenue. In this proof-of-concept study, we demonstrate the feasibility of rescuing distinct aberrant splicing patterns using tailored RNA-targeted approaches.

**Results:**

We focused on two disease-causing intronic pathogenic variants in the *CAPN3* gene (c.1193 + 30G > A and c.1354 + 5G > A), each leading to aberrant 5’ splice site selection and premature termination codons. Using a faithful cellular minigene model, we designed and evaluated two variant-specific corrective strategies: a splice-switching oligonucleotide (SSO) to block a gained cryptic donor site (c.1193 + 30G > A), which restored canonical transcript levels to approximately 75% of wild-type; and an engineered U1 snRNA with compensatory base substitutions to restore a weakened canonical 5’ splice site (c.1354 + 5G > A), which increased correct splicing from ~ 10% to nearly 60%.

**Conclusions:**

This work establishes a versatile therapeutic framework, providing compelling in vitro validation that precisely targeted RNA-based strategies can be successfully adapted to correct different types of splicing defects, offering a promising blueprint for the treatment of splicing-deficient genetic disorders.

**Supplementary Information:**

The online version contains supplementary material available at 10.1186/s13023-026-04451-x.

## Introduction

Limb-girdle muscular dystrophy type R1 (LGMDR1) is the most common subtype of limb-girdle muscular dystrophy, accounting for approximately 20%–30% of all cases, with a global prevalence of about 8.4 per million [1, 2]. Patients typically present in adolescence (6–18 years of age) with progressive weakness and atrophy of the shoulder and pelvic girdle muscles, leading to severe impairment of basic motor functions such as walking and standing [3]. Magnetic resonance imaging (MRI) often reveals fatty infiltration of bilateral hip and thigh muscle groups. Histopathological examination with hematoxylin and eosin (H&E) staining shows variability in fiber size, along with necrotic and regenerating fibers, while nicotinamide adenine dinucleotide (NADH) staining demonstrates disorganization of the myofibrillar network in some muscle fibers [4]. The disease carries a high disability rate, with approximately 80% of patients losing independent ambulation before the age of 40 and becoming wheelchair-dependent, imposing substantial physical, psychological, and economic burdens on both patients and their families [5]. However, to date, there remains a lack of specific therapies capable of delaying or halting disease progression in LGMDR1. Current clinical management is limited to symptomatic and supportive care, which falls far short of meeting the strong unmet clinical need. Notably, a defined subset of LGMDR1 cases is attributed to pathogenic variants that disrupt RNA splicing. For instance, in a cohort study encompassing 521 LGMDR1 patients, intronic pathogenic variants leading to aberrant splicing accounted for approximately 13.05% of all cases, underscoring the critical role of splicing dysregulation in disease pathogenesis [6].

Pre-mRNA splicing is a precisely orchestrated process essential for eukaryotic gene expression, requiring the accurate identification of exon-intron boundaries and the assembly of the spliceosome [7, 8]. This fidelity is maintained through a complex interplay of trans-acting splicing factors and cis-regulatory elements within the pre-mRNA [9, 10]. Central to the initiation of spliceosome assembly is the recognition of the 5’ splice site (5’ ss) by the U1 small nuclear ribonucleoprotein (U1 snRNP) [11]. The U1 snRNA, through complementary base-pairing, engages a consensus sequence spanning the exon-intron junction, primarily interacting with the last three nucleotides of the exon (positions − 3 to -1) and the first six nucleotides of the intron (+ 1 to + 6) [12]. This interaction ensures the precise demarcation of exons and facilitating the subsequent recruitment of additional spliceosome components. Pathogenic variants within the 5’ splice site can disrupt this essential base-pairing interaction, compromising U1 snRNP binding and frequently leading to aberrant splicing and disease pathogenesis [13–15]. To counteract these pathogenic variants, a therapeutic strategy has emerged involving the engineering of U1 snRNP components. By modifying the 5’ end of U1 snRNA to enhance complementarity to a mutated 5’ ss, binding affinity can be restored, thereby rescuing normal splicing patterns. This approach has demonstrated promising corrective efficacy across various disease models in vitro and in vivo, highlighting its potential as a targeted transcriptional therapy [16–19].

While engineered U1 snRNA is particularly suited for rescuing pathogenic variants that directly weaken the canonical 5’ splice site, a broader range of splicing defects originates from deep intronic pathogenic variants that create or activate cryptic splice sites. To correct such defects, splice-switching oligonucleotides (SSOs) represent a powerful alternative strategy. These short, synthetic, single-stranded nucleic acids are engineered with specific chemical modifications [20, 21]. Their primary mechanism involves precise base-pairing to target pre-mRNA sequences, thereby sterically blocking the binding of splicing machinery or regulatory proteins to cis-acting elements [22]. This modulation can redirect splicing patterns, correct aberrant splicing, and promote the production of functional protein isoforms. The clinical potential of SSOs is well-established, with several FDA-approved drugs for neuromuscular disorders, such as Nusinersen for spinal muscular atrophy (SMA) and Eteplirsen and Golodirsen for Duchenne muscular dystrophy (DMD) [23–25].

In this study, we focused on two LGMDR1 patients carrying distinct intronic pathogenic variants that were previously identified and characterized in our cohort [26]. Both variants were found to cause aberrant alternative 5’ splice site selection (A5SS), which introduces premature termination codons (PTCs) into the *CAPN3* transcript and leads to its degradation via the nonsense-mediated decay (NMD) pathway. We first constructed minigene vectors that successfully recapitulated the patient-specific aberrant splicing events in vitro. Utilizing this model, we then explored targeted therapeutic strategies tailored to the specific molecular defect of each pathogenic variant. For the c.1193 + 30G > A pathogenic variant (according to the transcript NM_000070.2), which creates a cryptic splice site, we designed splice-switching oligonucleotides (SSOs) to block this aberrant site and promote the use of the canonical one. For the c.1354 + 5G > A pathogenic variant (according to the transcript NM_000070.2), which weakens the canonical 5’ ss, we engineered a modified U1 small nuclear RNA (snRNA) to restore its recognition by the spliceosome. Our work demonstrates the application of variant-specific splice correction strategies and provides a framework for developing targeted therapies for splicing defects in LGMDR1.

## Methods

### Subjects

This study included patients from two Chinese families who received a diagnosis of limb-girdle muscular dystrophy recessive type 1 (LGMDR1) at Qilu Hospital of Shandong University during 2016–2017.

### Muscle pathology

Muscle biopsy specimens were obtained from both patients. Immediately after collection, the samples were snap-frozen in liquid nitrogen and stored at − 80 °C until analysis. Routine histopathological assessments were performed, which included hematoxylin and eosin (H&E) staining and nicotinamide adenine dinucleotide tetrazolium reductase (NADH-TR) staining.

### Whole exome sequencing and sanger sequencing

Peripheral blood samples were drawn from the two probands and their available family members for genetic analysis. Genomic DNA was extracted from these samples. For whole exome sequencing (WES), the DNA was sheared, purified, and processed for library construction. Target enrichment was carried out using a custom NimbleGen SeqCap EZ kit (Roche). The prepared libraries were then sequenced on an Illumina NextSeq500 platform.

Primary bioinformatics analysis involved aligning the obtained sequencing reads to the human reference genome (GRCh37/hg19). Variant calling and initial filtration were performed with NextGENe software (v2.4.1.2, SoftGenetics). Detected variants were annotated using public population and disease-specific databases. Analysis focused on variants present in the probands. We filtered out synonymous variants and common single nucleotide polymorphisms (SNPs) with a minor allele frequency (MAF) > 0.1% in general populations. The remaining rare, high-quality variants were prioritized as candidate pathogenic variants.

The potential impact of these candidate variants was evaluated using predictive in silico tools (including SIFT, PolyPhen-2, and CADD) and cross-referenced with databases such as OMIM, dbSNP, gnomAD, and ClinVar. Final variant classification was conducted in accordance with the guidelines established by the American College of Medical Genetics and Genomics (ACMG) [27, 28].

To confirm the WES findings, candidate variants were validated by Sanger sequencing in the probands and their parents. The relevant genomic regions were amplified by polymerase chain reaction (PCR). The resulting PCR products were then purified and sequenced on an Applied Biosystems 3500XL Genetic Analyzer.

### Minigenes and plasmids

To investigate the splicing patterns of *CAPN3*, two minigene constructs were engineered. The first construct contained a synthesized genomic fragment spanning exon 9, a partial sequence of intron 9, and exon 10 of the human *CAPN3* gene. The second construct contained a fragment spanning exon 10, a partial sequence of intron 10, and exon 11. All DNA fragments were synthesized by Sangon Biotech (Shanghai, China). Each fragment was subsequently cloned into a mammalian expression vector via homologous recombination using the ClonExpress^®^ II One Step Cloning Kit (Vazyme Biotech, China), placing it under the transcriptional control of a cytomegalovirus (CMV) promoter. Site-directed mutagenesis was then performed on these wild-type minigene plasmids to introduce the specific patient-derived variants, using the Mut Express^®^ II Fast Mutagenesis Kit V2 (Vazyme Biotech, China), thereby generating the corresponding mutant constructs for functional analysis. The primer sequences used for site-directed mutagenesis are presented in Table 1.

**Table 1:** Sequence information

Method	Name	Sequence (5’ – 3’)
SSO design	SSO1	CCCTTACCTTTCTGGGTCTTC
SSO design	SSO2	CCTTTCTGGGTCTTCCTGGGTTC
SSO design	SSO3	CTTCCCCACCCTTACCTTTCTGG
SSO design	SSO-Ctrl	CCTCTTACCTCAGTTACAATTTATA
qPCR	*CAPN3*-E9E10-Canonical-F	AGGATGGAGAGTTCTGGATGTC
qPCR	*CAPN3*-E9E10-Canonical-R	TGCAGATCTCCAACTTTGTGA
qPCR	*CAPN3*-E9E10-Total-F	ATGAGAAGGCCCGTCTGC
qPCR	*CAPN3*-E9E10-Total-R	TGCAGATCTCCAACTTTGTGA
qPCR	*CAPN3*-E10E11-Canonical-F	GCCGCAACTTCCCAGATACT
qPCR	*CAPN3*-E10E11-Canonical-R	CTCGTAGATGGCGAAGCCAA
qPCR	*CAPN3*-E10E11-Total-F	TGTCCTATGAGGATTTCATCTACCA
qPCR	*CAPN3*-E10E11-Total-R	CTCGTAGATGGCGAAGCCAA
qPCR	GAPDH-F	CAGAACATCATCCCTGCCTCTAC
qPCR	GAPDH-R	TTGAAGTCAGAGGAGACCACCTG
mutagenesis	+ 30G > A-F	CCCTTACCtTTCTGGGTCTTCCTGGGTTCTGG
mutagenesis	+ 30G > A-R	ACCCAGAAaGGTAAGGGTGGGGAAGAGAGGGG
mutagenesis	+ 5G > A-F	CAGGTGGaAGATGCTCTTGATGGGGGGAGGGT
mutagenesis	+ 5G > A-R	AGAGCATCTtCCACCTGGGAAGTTGCGGCAGC
mutagenesis	U1modified-F	TCATTTCCACCTggCAggggAgATACCATgATCAC
mutagenesis	U1modified-R	CTgCCAggTggAAATgAgATCTTgggCCTCTgCc

The wild-type human U1 snRNA expression plasmid was constructed as follows: the human U1 snRNA sequence was obtained from the study by Murphy et al. [29], chemically synthesized by Boshan Company, and cloned into the pcDNA3.1 vector (Invitrogen, Cat. No. V79520) by replacing the original sequence between MluI (ACGCGT) and NheI (GCTAGC) restriction sites (containing CMV enhancer, CMV promoter and T7 promoter) with the U1 snRNA coding sequence. The modified U1 snRNA expression vector targeting the *CAPN3* c.1354 + 5G > A pathogenic variant was generated by introducing four compensatory base substitutions into the 5’ end of the U1 snRNA sequence using the above-described wild-type U1 snRNA plasmid as the backbone, with the Mut Express^®^ II Fast Mutagenesis Kit V2 (Vazyme Biotech, China), as previously reported [30].

### Splice-switching oligonucleotides (SSOs)

All SSO sequences were designed following previously published guidelines [31, 32]. HPLC-purified SSOs with PS (phosphorothioate backbone) modifications and 2’-O-methylated modifications plus PS modifications were supplied by Sangon Biotech (Shanghai, China). All SSOs were dissolved in nuclease-free water for in vitro cell experiments.

### Cell culture and transfection

HEK293T cells were cultured in DMEM supplemented with 10% (vol/vol) fetal bovine serum (FBS) in 6-well plates. Transfection was performed using jetPRIME transfection reagent (Polyplus-transfection, France). Specifically, approximately 2 µg of minigenes or U1 snRNA expression plasmids were transfected per well. For splice-switching oligonucleotides (SSOs) treatment, cells were transfected with SSOs at a final concentration of 150 nM. Following each transfection, cells were incubated for 48 h prior to analysis.

### RNA isolation and quantitative PCR assay

Total RNA was extracted from HEK293T using FastPure Cell/Tissue Total RNA Isolation Kit V2 (Vazyme Biotech, China). Then, 1 µg of RNA was reverse transcribed into cDNA using HiScript II One Step RT-PCR Kit (Vazyme Biotech, China). To assess splicing patterns, quantitative PCR analysis was performed on synthesized cDNA templates with SYBR Green mix (Vazyme) on an Applied Biosystems QuantStudio 3 instrument. Two independent primer sets were designed for each minigene construct to quantify different transcript populations: for the canonical transcript-specific primers, the forward primer spanned the junction between two consecutive exons (exon 9-exon 10 for the c.1193 + 30G > A minigene; exon 10-exon 11 for the c.1354 + 5G > A minigene), and the reverse primer was located within the downstream exon. This primer set exclusively amplifies correctly spliced canonical transcripts; for the total transcript primers, the forward primer is located within the upstream exon, and the reverse primer is located within the downstream exon. This design allows amplification of all transcripts including the canonical transcript, the A5SS transcript, and any intron-retained transcripts. The relative expression of the canonical transcript was calculated using the 2 ^−ΔΔCt^ method with GAPDH as the endogenous reference gene. The ratio of canonical transcript to total transcript was calculated by dividing the GAPDH-normalized canonical transcript level by the GAPDH-normalized total transcript level. The detailed sequences of primers are presented in Table 1.

### RNA-seq and data analysis

Total RNA was isolated from HEK293T using TRIzol reagent (Invitrogen) according to the manufacturer’s protocol. Total RNA samples were reverse transcribed using HiScript II Q RT SuperMix for qPCR (R222-01, Vazyme). RNA-seq libraries were constructed using a two-step PCR approach. First round PCR reaction was performed in a total volume of 25 µL containing 12.5 µL 2×ChamQ SYBR Color qPCR Master Mix (Low ROX Premixed), 6.5 µL nuclease-free water, 2.5 µL each of first-round forward and reverse primers (1 µM), 1 µL cDNA template. PCR was carried out on a QuantStudio 3 system with the following program: initial denaturation at 94 ℃ for 3 min; 15 cycles of denaturing at 94 ℃ for 10 s, annealing at 60 ℃ for 15 s and elongation at 72 ℃ for 20 s; followed by a final extension at 72 ℃ for 10 min and hold at 4 ℃. The second round PCR was performed as follows: 15 µL 2×ChamQ SYBR Color qPCR Master Mix (Low ROX Premixed), 11 µL H2O, 0.5 µL Second-Round Primer-F (10 µM), 0.5 µL Second-Round Primer-R (10 µM), 3 µL Template (10 times diluted). The plate was sealed and PCR performed in a QuantStudio 3 system using the following program: 1 cycle of denaturing at 94 ℃ for 3 min, then 8 cycles of denaturing at 94 ℃ for 10 s, annealing at 60 ℃ for 15 s, elongation at 72 ℃ for 30 s, and a final extension at 72 ℃ for 10 min. Finally hold at 16 ℃. Amplicon products were purified using AMPure XP beads (Beckman Coulter, Brea, CA) according to the manufacturer’s instructions. Purified libraries were quantified, pooled in equimolar amounts, and subjected to paired-end sequencing on the Illumina NovaSeq 6000/X Plus platforms with a PE150 read length (Illumina, San Diego, CA). The primer sequences used for library construction are presented in Table 2. For sequencing data analysis, raw reads were processed using a custom MicroAS-seq bioinformatics pipeline specifically designed for targeted splicing variant analysis. The pipeline integrates adapter trimming, barcode demultiplexing, genome alignment, and transcript quantification steps. The complete pipeline code and detailed documentation are publicly available in the GitHub repository at https://github.com/PrinceWang2018/MicroAS-seq. Splicing patterns were visualized using the Integrative Genomics Viewer (IGV, version 2.16.0).

**Table 2:** The primer sequences used for RNA-seq libraries construction

Name	Sequence (5’ – 3’)
*CAPN3*_E10I10E11_Round1_F	TCGGCAGCGTCAGATGTGTATAAGAGACAGATCACGTGTCCTATGAGGATTTCATCTACCA
*CAPN3*_E10I10E11_Round1_R	CGTGGGCTCGGAGATGTGTATAAGAGACAGGTTTCGCTCGTAGATGGCGAAGCCAA
*CAPN3*_E9I9E10_ Round1_F	TCGGCAGCGTCAGATGTGTATAAGAGACAGATCACGTGGAAGGACTGGAGCTTTGTAG
*CAPN3*_E9I9E10_ Round1_R	CGTGGGCTCGGAGATGTGTATAAGAGACAGGTTTCGTGAAGCTTATCGGACTCCAGG
Round2_F	AATGATACGGCGACCACCGAGATCTACACAGCGCTAGTCGTCGGCAGCGTCAGATG
Round2_R	CAAGCAGAAGACGGCATACGAGATGTGAATATGTCTCGTGGGCTCGGAGATG

### Statistics and reproducibility

All quantitative data are presented as mean ± standard deviation (SD). All statistical analyses were performed using GraphPad Prism software (version 8.0). Statistical comparisons between two groups were performed using two-tailed unpaired Student’s t-test. Statistical significance was considered as *p* < 0.05. For RT-qPCR measurements following minigene transfection, experimental replicates refer to independent transfection experiments conducted on separate days with freshly prepared cells and plasmids. All RT-qPCR data were obtained from three independent transfection experiments. For RNA-seq splicing profiling, all analyses were performed using three independent transfection experiments.

## Results

### Minigene assays validate aberrant splicing of *CAPN3* induced by intronic pathogenic variants

The two LGMDR1 patients included in this study have been previously described in detail [26], and their variant information has been deposited in the LOVD database under accession numbers Individual #00474757 (c.1193 + 30G > A) and Individual #00474758 (c.1354 + 5G > A). The genetic sequencing and muscle pathology findings for these two patients are summarized below (Fig. 1A-D). As reported in our previous study, both intronic pathogenic variants lead to aberrant splicing of *CAPN3* in patient skeletal muscle samples. Specifically, the c.1193 + 30G > A pathogenic variant (according to the transcript NM_000070.2) causes the insertion of 31 nucleotides via alternative 5’ splice site (A5SS) selection, while the c.1354 + 5G > A pathogenic variant (according to the transcript NM_000070.2) results in a 40-nucleotide deletion, also through A5SS selection. To further test potential therapeutic interventions, we employed a minigene approach, a validated tool for studying splicing regulation. Two minigene constructs were generated: one containing *CAPN3* exon 9, a portion of intron 9, and exon 10; the other containing exon 10, a portion of intron 10, and exon 11(Fig. 1E, F). The detailed sequences of minigene are provided in the Supplementary Materials. The patient-derived pathogenic variants were subsequently introduced into the respective minigenes using site-directed mutagenesis. To verify that our minigene system could faithfully recapitulate the aberrant splicing patterns observed in the patients, we transfected the constructs into 293T cells. Splicing outcomes were analyzed by reverse transcription quantitative polymerase chain reaction (RT-qPCR) using two primer sets: one set amplifying the correctly spliced canonical transcript with the forward primer spanning the exon-exon junction and the reverse primer located within the downstream exon, and another set amplifying all possible transcripts from the minigene with primers positioned at the ends of the two flanking exons (Fig. 1E, F). The results demonstrated that the c.1193 + 30G > A pathogenic variant reduced the expression of the canonical transcript to 34.10%±2.34% of the wild-type level, and the proportion of the canonical transcript among total transcripts decreased to 10.09%±3.96% of the wild-type ratio (Fig. 1G, H). Similarly, the c.1354 + 5G > A pathogenic variant markedly diminished the expression of the canonical transcript to 3.44%±0.16% of the wild-type level, and its proportion among total transcripts was reduced to 11.80%±0.75% of the wild-type ratio (Fig. 1I, J).

**Fig. 1:**
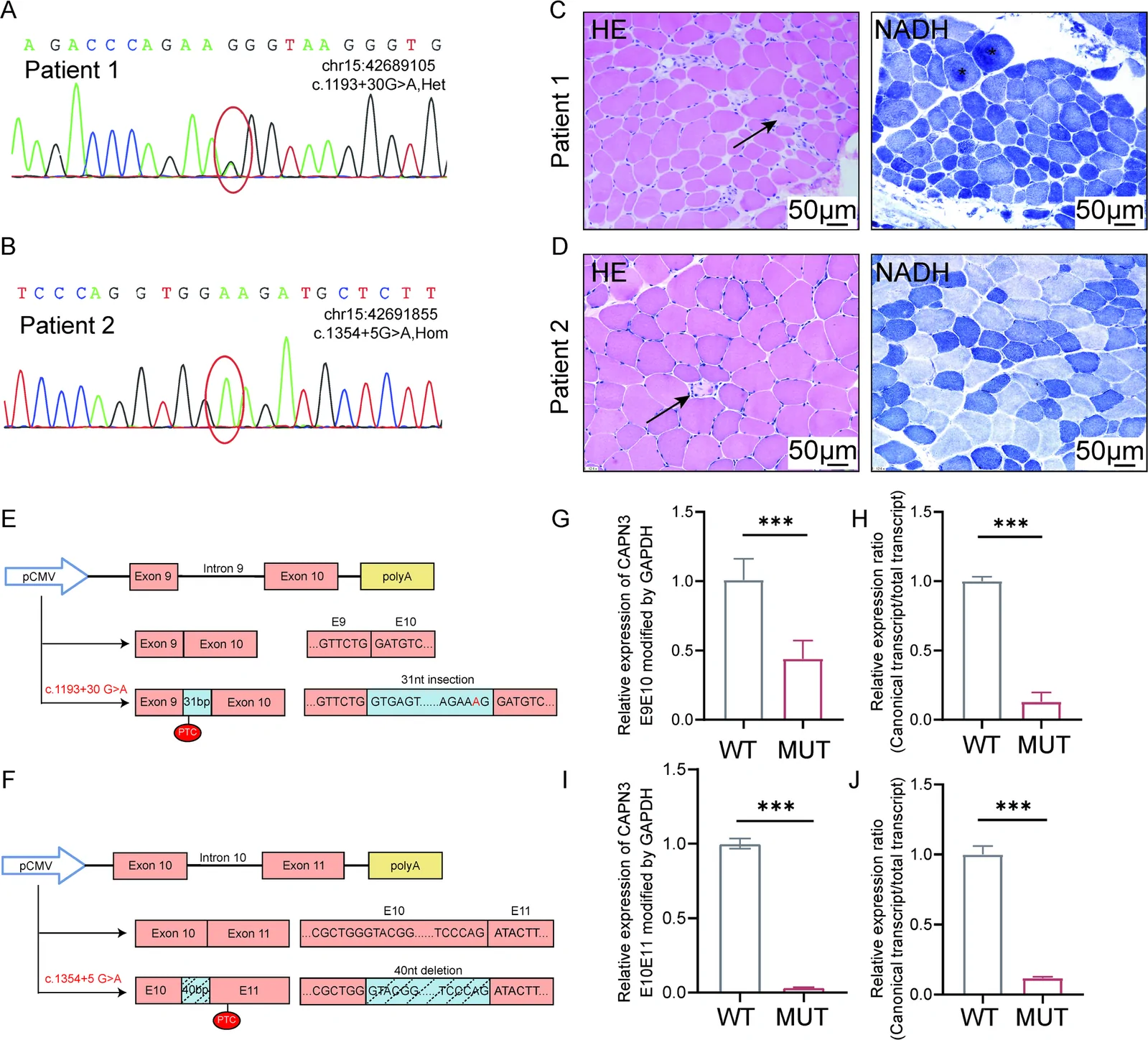
Minigene construction based on patient clinical data. (**A-B**) Sanger analysis of two LGMDR1 patients, variants indicated by red ovals. (**C-D**) Muscle pathology of the patients. HE staining reveals variation in muscle fiber size in both patients, with occasional necrotic fibers (arrowheads). NADH staining shows disorganized myofibrillar network in some muscle fibers of patient 1 (asterisks). (**E-F**) Schematic representation of the *CAPN3* minigene constructs and transcript products with intron 9 variants (c.1193 + 30G > A) (**E**) and intron 10 variant (c.1354 + 5G > A) (**F**). PTC, premature termination codon. (**G-J**) RT-qPCR analysis of relative expression of *CAPN3* canonical transcript and ratio of *CAPN3* canonical transcript to total transcript in HEK293T transfected with *CAPN3* minigene harboring c.1193 + 30G > A (**G-H**) and c.1354 + 5G > A (**I-J**). Data are presented as mean ± SD from three independent transfection experiments. Statistical significance was determined by two-tailed unpaired Student’s t-test. ***p < 0.001

### Splice-switching oligonucleotides mediated correction of aberrant splicing induced by the c.1193 + 30G > A pathogenic variant

Splice-switching oligonucleotides (SSOs) serve as potent tools for modulating pre-mRNA splicing, with several splice‑switching oligonucleotides-based therapeutics already approved for clinical use. The c.1193 + 30G > A pathogenic variant activates a cryptic splice donor site that competes with the canonical splice site for splicing factor binding, leading to a 31‑nucleotide insertion. We hypothesized that SSOs targeting the region adjacent to this aberrant splice donor site could block its usage, thereby restoring canonical splicing. Accordingly, we designed three candidate SSOs along with a control SSO that does not share complementarity to the minigene sequence (Fig. 2A). The detailed sequences of SSOs are presented in Table 1. To enhance stability and resist nuclease degradation, all SSOs were phosphorothioate‑modified. Co‑transfection of the minigene with each SSO into HEK293T cells was performed, and the proportion of canonical transcripts relative to total transcripts was quantified by RT‑qPCR. Results showed that two of the three SSOs increased the percentage of canonical splicing compared with the control SSO. Among them, SSO2 exhibited the highest efficacy, restoring canonical splicing to approximately 16.12%±2.97% of the wild‑type level (Fig. 2B).

**Fig. 2:**
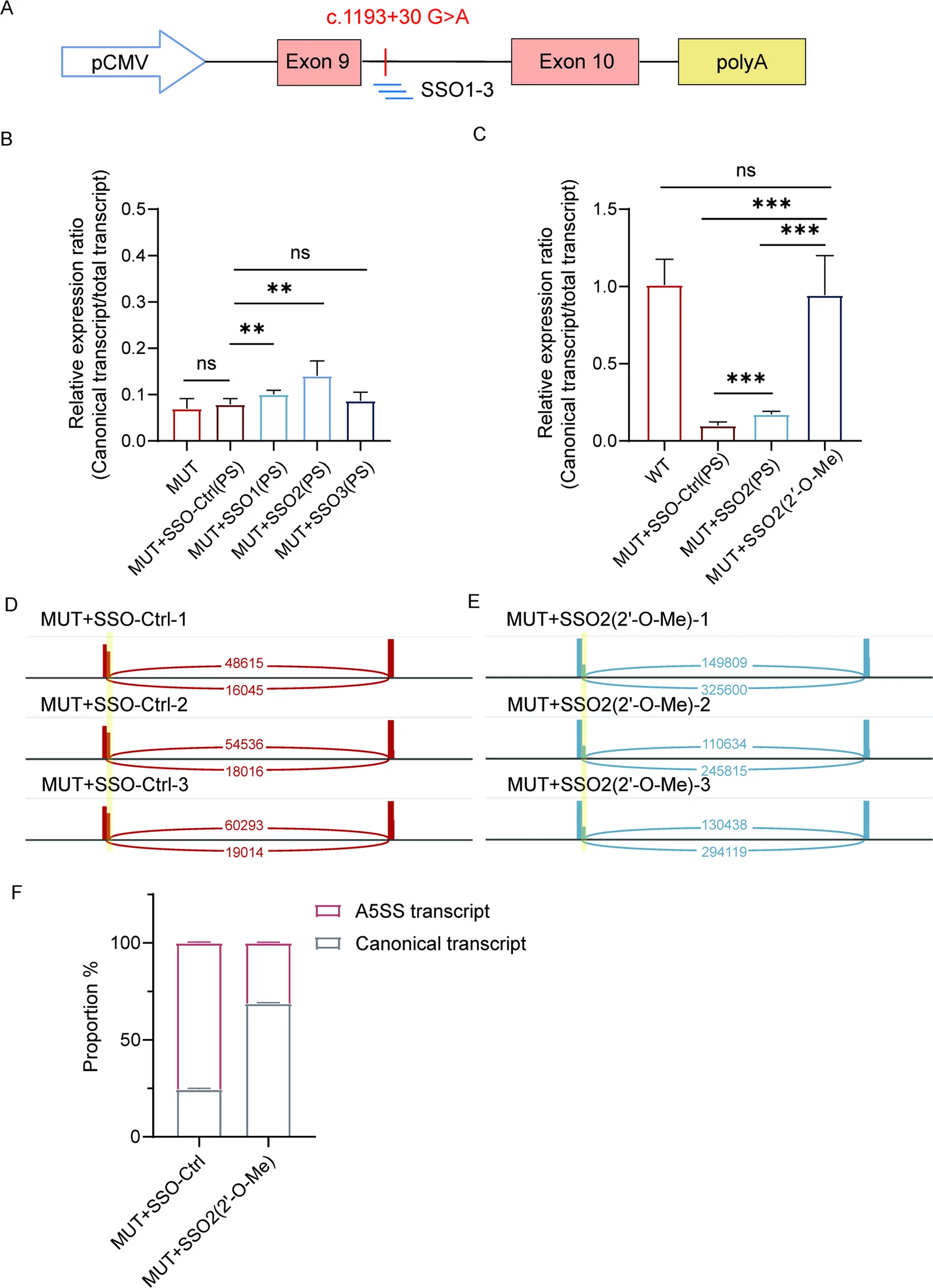
Splice correction of the c.1193 + 30G > A variant using SSOs. (**A**) Diagram of the targeting locations of SSOs on the minigene for the *CAPN3* c.1193 + 30G > A variant. (**B-C**) RT-qPCR analysis of the ratio of *CAPN3* canonical transcript to total transcript in HEK293T cells transfected with wild-type or c.1193 + 30G > A mutant minigenes, without or with co‑transfection of different SSOs. Data are presented as mean ± SD from three independent transfection experiments. Statistical significance was determined by two-tailed unpaired Student’s t-test. ns, not significant; **p < 0.01; ***p < 0.001. (**D-E**) Sashimi plots depicting the alternative 5’ splice site selection of *CAPN3* in HEK293T cells transfected with the minigene and treated with either control SSO (red) or 2’-O-methylated SSO2. The 31 nt sequence inserted from the 5’ end of intron 9 in the alternative 5’ splice site (A5SS) transcript is highlighted in yellow. (**F**) Stacked bar chart representing the relative proportions of individual transcripts. Data are from three independent transfection experiments

### 2’-O-methylated modification further enhances SSO-mediated splicing correction efficiency

To further enhance efficacy, we introduced 2’-O-methylated modifications in addition to the phosphorothioate backbone. This modification strategy has been previously reported to strengthen the binding affinity of SSOs to their target sequences. The results demonstrated that the 2’-O-methylated modification significantly improved splicing correction efficiency, increasing the proportion of canonical transcripts to approximately 74.59%±16.54% of the wild-type level. This represents a 5-fold enhancement compared to the efficacy of the phosphorothioate-modified SSO2 (Fig. 2C). RT-PCR gel electrophoresis and Sanger sequencing confirmed the identity of all splicing products from the c.1193 + 30G > A minigene experiment (Supplementary Fig. 1A). Two major bands were consistently observed across all samples: a lower band corresponding to the canonical transcript (theoretical size 99 bp) and an upper band corresponding to the A5SS transcript containing a 31 nt intronic insertion (theoretical size 130 bp). Both bands were excised, cloned into T-vectors, and Sanger sequenced to confirm their exact sequences. Two additional faint bands were observed above the A5SS band: a band at approximately 300 bp corresponding to full intron 9 retention, which is attributed to the strong transcriptional activity of the CMV promoter leading to overproduction of pre-mRNA transcripts that exceed the processing capacity of the cellular splicing machinery, resulting in incomplete splicing; and a non-specific band between 200 and 250 bp that showed no consistent difference between groups. Therefore, both bands were excluded from quantitative analysis. The percent spliced-in (PSI) values calculated from gel band intensities were highly consistent with the RT-qPCR results, visually confirming that SSO treatment effectively corrected the aberrant splicing event and restored the expression of the canonical transcript. While RT-qPCR can assess the relative proportion of canonical transcripts, it does not provide absolute quantification of individual transcript expression levels or a definitive evaluation of the SSO’s ability to correct the aberrant splicing event. Therefore, we performed RNA-seq for detailed analysis. The RNA-seq data revealed that in the SSO control group, canonical transcripts accounted for only 24.54%±0.40% of all transcripts, while transcripts containing the A5SS (alternative 5’ splice site) event constituted 75.46%±0.40% (Fig. 2D, F). This indicates that the novel splice site generated by the pathogenic variant is more readily recognized and utilized by the spliceosome than the canonical site. However, upon treatment with the blocking SSO, the proportion of canonical transcripts increased to 68.91%±0.32% (Fig. 2E, F), which is 2.80 times that of the control group. These results confirm that our designed SSO can efficiently correct aberrant splicing in this in vitro model.

### Therapeutic correction of aberrant 5’ splice site selection in *CAPN3* intron 10 caused by the c.1354 + 5G > A pathogenic variant using engineered U1 snRNA

Recognition of the splice donor site relies on complementary base pairing between the 5’ end of U1 snRNA and the nine nucleotides in the exon-intron junction. In the wild‑type sequence, U1 snRNA exhibits perfect complementarity to six bases spanning the exon 10/intron 10 of *CAPN3*. However, the c.1354 + 5G > A pathogenic variant reduces the complementary stretch to only five bases, with the last four bases being mismatched (Fig. 3A). This significantly impairs recognition efficiency, leading to the inactivation of the canonical splice site and the consequent activation of a cryptic splice site located 40 bp upstream. To restore recognition of the canonical splice site by the spliceosome, we designed a modified U1 snRNA by substituting four nucleotides to achieve full complementarity with the mutated sequence (Fig. 3B). Co‑transfection of the minigene construct together with the modified U1 snRNA plasmid into HEK293T cells resulted in the recovery of the canonical transcript to 110.04% ± 5.03% of the wild‑type level, while its relative proportion reached 98.20% ± 6.60% (Fig. 3C, D). Notably, co‑transfection with wild‑type U1 snRNA failed to correct the aberrant splicing, indicating that the rescue effect was sequence‑specific rather than a consequence of U1 snRNA overexpression. RT-PCR gel electrophoresis and Sanger sequencing validated the splicing products from the c.1354 + 5G > A minigene experiment (Supplementary Fig. 1B). Two bands were consistently observed across all samples: the lower band corresponding to the canonical transcript (theoretical size 166 bp), and the upper band at approximately 522 bp corresponding to full intron 10 retention. This intron retention event is attributed to the strong transcriptional activity of the CMV promoter leading to overproduction of pre-mRNA transcripts that exceed the processing capacity of the cellular splicing machinery, resulting in incomplete splicing. Given that this intron retention event was not detected in patient muscle samples [26] and represents an artifact of the minigene overexpression system, it was not included in our quantitative analysis. The intensity of the canonical band was markedly reduced in the mutant minigene group compared to the wild-type group, and co-transfection with the engineered U1 snRNA fully restored its abundance, consistent with the RT-qPCR results. No distinct band corresponding to the predicted A5SS transcript (theoretical size 126 bp) was visible on the agarose gel. We attribute this discrepancy to the nonsense-mediated mRNA decay (NMD) pathway. The 40 nt deletion in the A5SS transcript causes a frameshift and introduces a premature termination codon which targets the aberrant transcript for rapid degradation in mammalian cells. RNA‑seq analysis further confirmed these findings and provided a comprehensive view of the splicing landscape altered by the c.1354 + 5G > A variant. In the untreated mutant group, the major aberrant transcript was the previously identified alternative 5’ splice site (A5SS) transcript containing a 40 nt deletion from the 3’ end of exon 10, which accounted for 81.58% ± 0.93% of all transcripts. In addition to this predominant transcript, two low-abundance aberrant splicing events were detected in the mutant group and were almost undetectable in the wild-type group. Both minor aberrant transcripts utilized the same cryptic 5’ splice site as the major A5SS transcript (45 nucleotides upstream of the canonical donor site) but employed alternative downstream 3’ splice sites located 23 bp and 82 bp downstream of the canonical acceptor site, respectively. Together, these two additional transcripts constituted only 9.19% ± 0.64% of total transcripts. For clarity of presentation, only the canonical transcript and the major A5SS transcript are shown in the sashimi plots (Fig. 4A), while the “other transcript” category in the stacked bar chart (Fig. 4B) represents the sum of these two low-abundance aberrant splicing events. In the untreated mutant group and wild‑type U1 snRNA co‑transfection group, the proportions of the canonical transcript were 9.23% ± 0.29% and 9.60% ± 0.17%, respectively (Fig. 4B). In contrast, co‑transfection with the modified U1 snRNA resulted in a dramatic shift in splicing patterns, with the proportion of the canonical transcript increasing to 57.05% ± 0.63%. Notably, the engineered U1 snRNA not only significantly reduced the proportion of the major A5SS transcript (from 81.58% ± 0.93% to 38.48% ± 0.44%), but also effectively decreased the levels of both additional low-abundance aberrant transcripts (from 9.19% ± 0.64% to 4.47% ± 0.27%), demonstrating its broad efficacy in correcting all splicing abnormalities caused by the c.1354 + 5G > A variant.

**Fig. 3:**
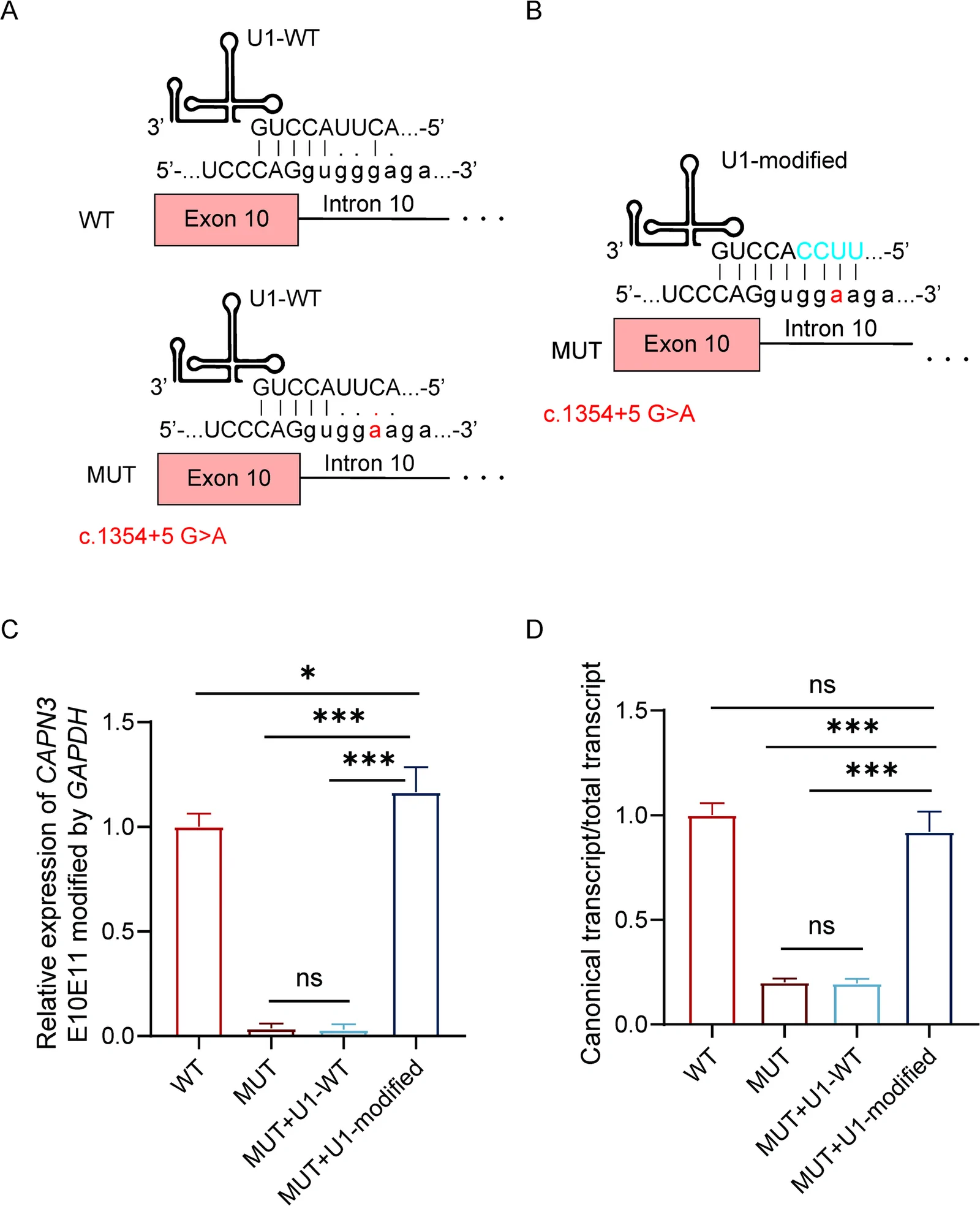
Development of an engineered U1 snRNA to target the *CAPN3* c.1354 + 5G > A pathogenic variant. (**A**) Mechanism by which the *CAPN3* pathogenic variant c.1354 + 5G > A impairs recognition of the splice donor site by wild-type U1 snRNA (U1-WT). The G-to-A transition reduces complementarity between U1 snRNA and the exon 10 donor site from 6 to 5 base pairs. The mutated nucleotide is highlighted in red. (**B**) Schematic of engineered U1 snRNA designed to bind the splice donor site of *CAPN3* exon 10/intron 10. Nucleotides altered within the U1 snRNA are highlighted in blue. (**C–D**) RT‑qPCR analysis of *CAPN3* transcript expression in HEK293T cells transfected with wild-type or c.1354 + 5G > A mutant minigenes, without or with co‑transfection of wild‑type or modified U1 snRNAs. (**C**) Relative expression of the canonical transcript. (**D**) Ratio of canonical to total transcripts. Data are presented as mean ± SD from three independent transfection experiments. Statistical significance was determined by two-tailed unpaired Student’s t-test. ns, not significant; *p < 0.05; ***p < 0.001

**Fig. 4:**
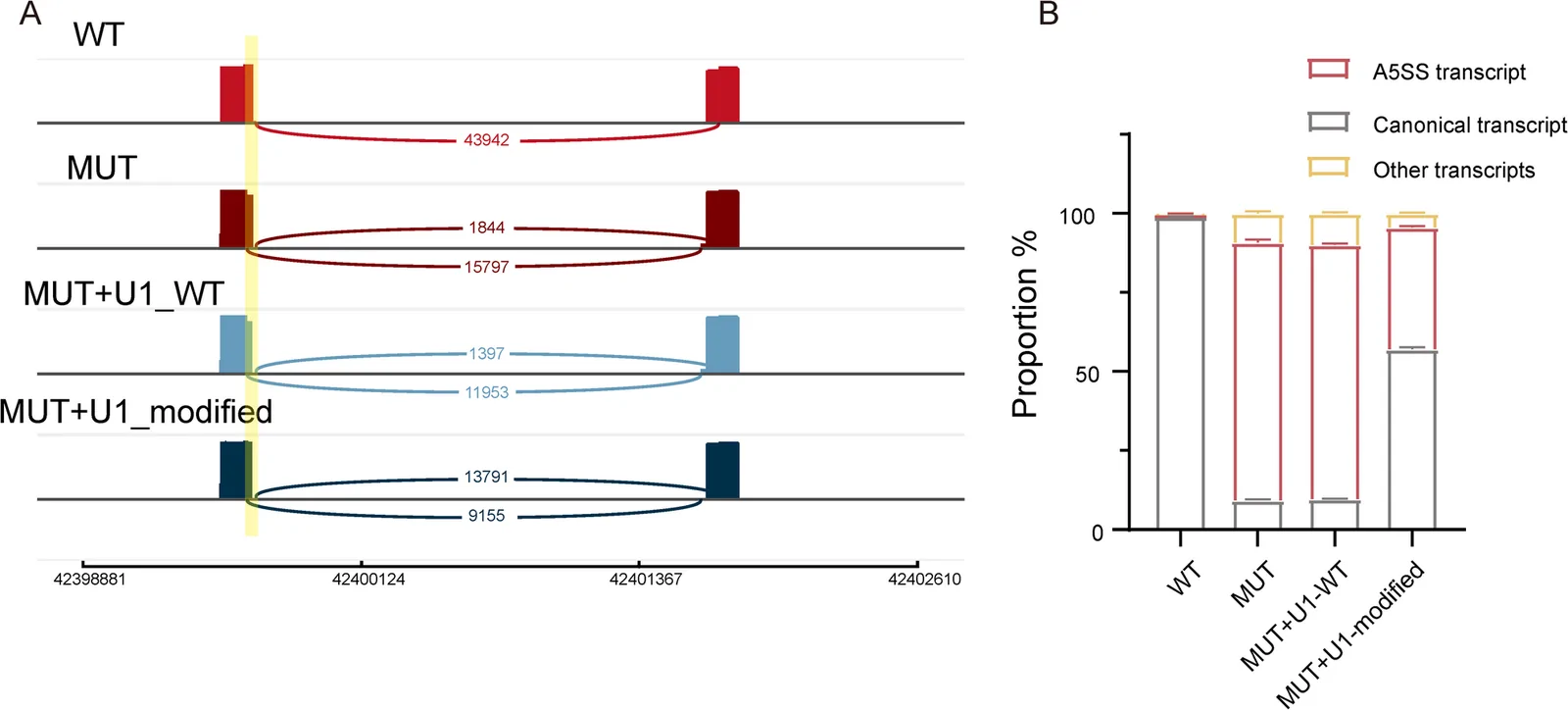
Validation of the efficacy of engineered U1 snRNA in rescuing aberrant splicing induced by the c.1354 + 5G > A pathogenic variant using RNA-seq. (**A**) Sashimi plots depicting the alternative 5’ splice site selection of *CAPN3* in HEK293T cells transfected with wild-type or c.1354 + 5G > A mutant minigenes, without or with co‑transfection of wild‑type or modified U1 snRNAs. The 40 nt sequence deleted from the 3’ end of exon 10 in the alternative 5’ splice site (A5SS) transcript is highlighted in yellow. The numbers indicate the read counts supporting each splice junction. (**B**) Stacked bar chart representing the relative proportions of individual transcripts. The “Other transcripts” category represents the sum of two low-abundance aberrant splicing events that share the same cryptic 5’ splice site as the major A5SS transcript but utilize alternative downstream 3’ splice sites. Data are from three independent transfection experiments

## Discussion

This study demonstrates the feasibility of variant-specific splice correction, using two distinct intronic variants in *CAPN3* as illustrative cases. By employing a minigene system that faithfully recapitulated the patient-specific aberrant splicing events, we successfully rescued canonical splicing for the c.1193 + 30G > A pathogenic variant using a splice-switching oligonucleotide (SSO) and for the c.1354 + 5G > A pathogenic variant using an engineered U1 snRNA. These findings provide a robust proof-of-concept for a mechanism-guided, RNA-targeted therapeutic framework applicable to splicing defects.

The application of these two distinct approaches is dictated by the fundamentally different mechanisms through which the intronic pathogenic variants disrupt splicing. The c.1193 + 30G > A pathogenic variant creates a de novo cryptic 5’ splice site (5’ ss) that outcompetes the adjacent canonical site. In this scenario, an SSO designed to bind across the aberrant site acts through steric hindrance, blocking the access of the splicing machinery and thereby redirecting spliceosome assembly to the canonical donor site. In contrast, the c.1354 + 5G > A pathogenic variant disrupts the canonical 5’ ss by reducing its complementarity to U1 snRNA, resulting in its failure to be recognized and the engagement of an upstream cryptic site. Simply blocking this cryptic site with an SSO would likely be insufficient, as the impaired canonical site would remain unrecognizable, potentially leading to the utilization of other aberrant sites or exon skipping. Therefore, a compensatory strategy that restores molecular recognition—engineering the U1 snRNA to regain perfect complementarity to the mutated sequence—was necessary and proved effective. Therefore, for splicing-disrupting pathogenic variants, the choice of therapeutic strategy must be dictated by the specific molecular mechanism underlying the defect.

Our findings also highlight the critical importance of specific nucleotides within the 9-base 5’ ss consensus. For the c.1193 + 30G > A pathogenic variant, the created cryptic site and the canonical site both share 7-base complementarity with wild-type U1 snRNA. The competitive advantage of the cryptic site likely arises because the pathogenic variant changes the − 2 position (relative to the exon-intron junction) from a U to an A, establishing a canonical Watson-Crick base pair with U1 snRNA, whereas the wild-type sequence at this position does not. Conversely, the + 5 position, mutated in our second case (c.1354 + 5G > A), is highly conserved, and alterations here are frequently pathogenic, as they severely compromise U1 snRNP binding, often leading to complete abolition of the canonical site usage, as observed here [33–35].

Beyond strategy selection, we investigated the impact of chemical modifications on SSO efficacy. While a phosphorothioate (PS)-backbone-modified SSO provided apparent correction, restoring canonical transcripts to ~ 16% of wild-type levels, the incorporation of an additional 2’-O-methyl (2’-OMe) modification dramatically enhanced efficiency by approximately 5-fold, achieving near-wild-type levels (~ 75%). This aligns with prior reports that 2’-OMe modifications improve binding affinity and nuclease resistance, underscoring that optimizing oligonucleotide chemistry is crucial for maximizing therapeutic potential [36, 37].

Accurately quantifying the splicing outcomes was essential, particularly as both pathogenic variants induce what we term “micro-splicing events”—small insertions or deletions of 31 and 40 nucleotides, respectively. Such subtle changes are challenging to detected by conventional RT-PCR and gel electrophoresis. To overcome this, we employed a qPCR approach using junction-spanning primers for the canonical product and primers in the flanking exons for total transcripts, coupled with validation by RNA-seq. This combined strategy provided robust, quantitative assessment of these minor splicing alterations and the efficacy of our corrective interventions.

Our study has several limitations. Firstly, the use of a minigene model in HEK293T cells, while validated, cannot fully replicate the complex splicing environment of human skeletal muscle. The lack of patient-derived cells (e.g., fibroblasts or myoblasts) for confirmation is a notable constraint. Secondly, our minigene constructs only included minimal portions of introns 9 and 10, which may not capture all potential splicing events that could occur in the context of full-length introns. To address this concern, we performed comprehensive splice site prediction analysis using SpliceAI. Notably, all predicted splicing events, including the low-confidence ones, were located entirely within the regions included in our minigene constructs. Nevertheless, given the inherent complexity of splicing regulation, we cannot rule out the possibility of unpredicted splicing events occurring in the intronic regions not included in our minigene constructs. Thirdly, both therapeutic strategies carry inherent off-target risks that require systematic evaluation. While the engineered U1 snRNA showed high specificity in our model, it may potentially bind to non-target pre-mRNAs with partial complementarity to its modified 5’ tail, leading to unintended global splicing alterations. Similarly, splice-switching oligonucleotides (SSOs) are susceptible to sequence-dependent off-target effects, primarily through cross-hybridization with non-target RNAs containing short contiguous complementary regions, which can induce aberrant splicing regulation or RNase H-mediated degradation of unrelated transcripts [38]. For both therapeutic modalities, comprehensive transcriptome-wide analysis in relevant muscle cell types is required to fully assess potential off-target effects on global splicing and gene expression [39]. Additionally, the threshold of splicing correction needed to yield a clinically meaningful phenotypic improvement in LGMDR1 remains undefined and is a critical question for future in vivo studies. Finally, the principal challenge for clinical translation is delivery. For SSOs, conjugates for example peptide-conjugated phosphorodiamidate morpholino oligomers (PMOs) have proven effective for muscle delivery in other disorders [40, 41]. For U1 snRNA, adeno-associated virus (AAV) vectors are the primary vehicle for in vivo gene therapy, but they face hurdles including pre-existing immunity, packaging limitations, and potential safety concerns. Given these challenges, lipid nanoparticle (LNP) systems represent a viable non-viral alternative for delivering U1 snRNA-based therapeutics, building on their proven success in delivering other RNA-based drugs [42, 43].

Future research should focus on validating these strategies in more physiologically relevant models, such as patient-derived induced pluripotent stem cell (iPSC)-differentiated myotubes or humanized mouse models of LGMDR1. In vivo studies will be indispensable for evaluating biodistribution, long-term efficacy, safety, and the functional rescue of the calpain-3 protein and muscle pathology. Furthermore, transcriptomic profiling in animals treated with the engineered U1 snRNA or SSO2 will be crucial to confirm target specificity and rule out significant off-target splicing alterations for both therapeutic approaches.

In conclusion, we have delineated and validated a mechanism-guided framework for correcting aberrant splicing, exemplified by two distinct intronic pathogenic variants in *CAPN3*. The core principles—blocking a gained-function cryptic site or restoring recognition of a weakened canonical site—constitute a versatile therapeutic toolkit. Although demonstrated in a specific disease context, this framework is directly applicable to a broad subset of genetic disorders driven by similar splicing defects. This work thus establishes a generalizable conceptual and methodological blueprint for the development of RNA-targeted splice-correction therapies.

## Supplementary Information

Below is the link to the electronic supplementary material.

Supplementary Material 1: Supplementary Fig. 1 RT-PCR gel electrophoresis and Sanger sequencing validated the splicing products from minigene experiment. (A) c.1193 + 30G > A minigene experiment. Left panel: RT-PCR products separated on a 2.5% agarose gel. Two major bands were detected: the lower band corresponds to the canonical transcript (99 bp) and the upper band corresponds to the alternative 5’ splice site (A5SS) transcript (130 bp, containing a 31 nt intronic insertion). Lanes represent samples from HEK293T cells transfected with wild-type minigene (WT), or mutant minigene co-transfected with phosphorothioate (PS) control SSO (MUT + SSO-Ctrl(PS)), PS-modified SSO2 (MUT+SSO2(PS)), or 2’-O-methylated (2’-O-Me) SSO2 (MUT+SSO2(2’-O-Me)). The percent spliced-in (PSI) values of the canonical transcript are indicated below each lane, calculated as: PSI = (intensity of WT band) / (intensity of WT band + intensity of A5SS band). Right panel: Sanger sequencing validation of the excised bands. The top two chromatograms show sequences from the A5SS band, with the 31 nt intronic insertion highlighted by red boxes and the c.1193 + 30G > A pathogenic variant site marked by yellow shading. The bottom chromatogram shows the sequence from the WT band, demonstrating the correct junction between exon 9 and exon 10. (B) c.1354 + 5G > A minigene experiment. Left panel: RT-PCR products separated on a 2.5% agarose gel. Two bands were consistently observed: the lower band corresponding to the canonical transcript (166 bp), and the upper band at approximately 522 bp corresponding to full intron 10 retention. Lanes represent samples from HEK293T cells transfected with wild-type minigene (WT), mutant minigene alone (MUT), mutant minigene co-transfected with wild-type U1 snRNA (MUT+U1-WT), or mutant minigene co-transfected with engineered U1 snRNA (MUT+U1-modified). Right panel: Sanger sequencing validation of the excised canonical band, demonstrating the correct junction between exon 10 and exon 11. All images are representative of three independent transfection experiments

Supplementary Material 2

Supplementary Material 3

Supplementary Material 4

Supplementary Material 5

Supplementary Material 6

## Data Availability

Data available on request from the authors. All data generated in this study was included in this published article and its supplementary files.

## Abbreviations

CAPN3: Calpain 3
LGMDR1: Limb-Girdle Muscular Dystrophy R1
SSO: Splice-Switching Oligonucleotide
HE: Hematoxylin and Eosin
NADH: Nicotinamide Adenine Dinucleotide
U1 snRNP: U1 small nuclear ribonucleoprotein
U1snRNA: U1 small nuclear RNA
A5SS: Alternative 5’ splice site
CMV: Cytomegalovirus
RT-qPCR: Reverse Transcription Quantitative Polymerase Chain Reaction
PS: Phosphorothioate
2’-OMe: 2’-O-methyl
