# Supplementary material for: Correction of aberrant splicing caused by intronic CAPN3 pathogenic variants using RNA-targeted therapeutic strategies in limb-girdle muscular dystrophy type R1
Source: Orphanet J Rare Dis. 2026 Jun 13;21:292. doi: 10.1186/s13023-026-04451-x (PMC13495512; doi:10.1186/s13023-026-04451-x)
Supplement: Supplementary file 1 [file 13023_2026_4451_MOESM2_ESM.docx]

LOVD v.3.0 - Leiden Open Variation Database
Online gene-centered collection and display of DNA variants

Home News FAQ Documentation Download Contact Developers

LOVD 3.0 LOVD 2.0 Public list of LOVD installations Search for a variant Our list of Locus Specific Databases

Query all public LOVD installations

Query all public LOVD instances:

hg19 / GRCh37 | chr15:g.42689105G>A | Search

Examples: Precise: chr15:g.40699840C>T, Range: chr13:32936732-32936735.
Instead of "chr15", you can also use a reference sequence, e.g., NC_000015.9:g.40699840C>T. The given build will then be derived from your input.

LOVDs currently support only one genome build; if no results are found, you may want to repeat your query using a different genome build.
LOVD contains for hg18 ~2K unique variants, hg19 ~3.0M unique variants, and hg38 ~1.4M.
This service queries the variant's location, i.e. results of other variants on the same location will show as well. When searching using a ranged variant in HGVS format, only variants exactly matching that range will be returned. When searching using a range (2nd example above), all variants within that range will be returned (to a max of 50).

CAPN3 **NM_000070.2:c.1193+30G>A** Affects function / **Not classified**
https://databases.lovd.nl/shared/ *Variant location matches your query exactly*

When using or discussing LOVD please refer to:
Fokkema IF, Kroon M, López Hernández JA, Asscheman D, Lugtenburg I, Hoogenboom J, den Dunnen JT. *The LOVD3 platform: efficient genome-wide sharing of genetic variants.* Eur J Hum Genet (2021). https://doi.org/10.1038/s41431-021-00959-x.

LOVD has received funding from the European Community's Seventh Framework Programme (FP7/2007-2013) under grant agreement nº 200754 - the GEN2PHEN project.
©2004-2026 Leiden University Medical Center, Netherlands
Ivo F.A.C. Fokkema, PhD & Prof. Johan T. den Dunnen, PhD

LOVD v.3.0 - Leiden Open Variation Database
Online gene-centered collection and display of DNA variants

Home News FAQ Documentation Download Contact Developers

LOVD 3.0 LOVD 2.0 Public list of LOVD installations Search for a variant Our list of Locus Specific Databases

Query all public LOVD installations

Query all public LOVD instances:

hg19 / GRCh37 | chr15:g.42691855G>A | Search

Examples: Precise: chr15:g.40699840C>T, Range: chr13:32936732-32936735.
Instead of "chr15", you can also use a reference sequence, e.g., NC_000015.9:g.40699840C>T. The given build will then be derived from your input.

LOVDs currently support only one genome build; if no results are found, you may want to repeat your query using a different genome build.
LOVD contains for hg18 ~2K unique variants, hg19 ~3.0M unique variants, and hg38 ~1.4M.
This service queries the variant's location, i.e. results of other variants on the same location will show as well. When searching using a ranged variant in HGVS format, only variants exactly matching that range will be returned. When searching using a range (2nd example above), all variants within that range will be returned (to a max of 50).

CAPN3 **NM_000070.2:c.1354+5G>A** Affects function / **Not classified**
https://databases.lovd.nl/shared/ *Variant location matches your query exactly*

When using or discussing LOVD please refer to:
Fokkema IF, Kroon M, López Hernández JA, Asscheman D, Lugtenburg I, Hoogenboom J, den Dunnen JT. *The LOVD3 platform: efficient genome-wide sharing of genetic variants.* Eur J Hum Genet (2021). https://doi.org/10.1038/s41431-021-00959-x.

LOVD has received funding from the European Community's Seventh Framework Programme (FP7/2007-2013) under grant agreement nº 200754 - the GEN2PHEN project.
©2004-2026 Leiden University Medical Center, Netherlands
Ivo F.A.C. Fokkema, PhD & Prof. Johan T. den Dunnen, PhD
