# Supplementary material for: Correction of aberrant splicing caused by intronic CAPN3 pathogenic variants using RNA-targeted therapeutic strategies in limb-girdle muscular dystrophy type R1
Source: Orphanet J Rare Dis. 2026 Jun 13;21:292. doi: 10.1186/s13023-026-04451-x (PMC13495512; doi:10.1186/s13023-026-04451-x)
Supplement: Supplementary file 3 [file 13023_2026_4451_MOESM4_ESM.docx]

Supplementary sequences

Minigene-E9I9E10-WT

5’-

GATGAGAAGGCCCGTCTGCAGCACCAGGTCACTGAGGATGGAGAGTTCTGGTGAGTCCAGAACCCAGGAAGACCCAGAAGGGTAAGGGTGGGGAAGAGAGGGGAAATCTCAGACCTCAGTCCCCAGCTAAGGTTATCAGATTCCAGCCCTGAACTATTTTATTATTGTGCTGTGTTAGTCCTGGGGTCTTCCGTTCCCAGCCCTCCTCACCTGCTCCCATATGGCTCTCTCTCTTCTTCCAACCTCTCAGGATGTCCTATGAGGATTTCATCTACCATTTCACAAAGTTGGAGATCTGCA-3’

Minigene-E9I9E10-MUT

5’-

GATGAGAAGGCCCGTCTGCAGCACCAGGTCACTGAGGATGGAGAGTTCTGGTGAGTCCAGAACCCAGGAAGACCCAGAAAGGTAAGGGTGGGGAAGAGAGGGGAAATCTCAGACCTCAGTCCCCAGCTAAGGTTATCAGATTCCAGCCCTGAACTATTTTATTATTGTGCTGTGTTAGTCCTGGGGTCTTCCGTTCCCAGCCCTCCTCACCTGCTCCCATATGGCTCTCTCTCTTCTTCCAACCTCTCAGGATGTCCTATGAGGATTTCATCTACCATTTCACAAAGTTGGAGATCTGCA-3’

Minigene-E10I10E11-WT

5’-

GATGTCCTATGAGGATTTCATCTACCATTTCACAAAGTTGGAGATCTGCAACCTCACGGCCGATGCTCTGCAGTCTGACAAGCTTCAGACCTGGACAGTGTCTGTGAACGAGGGCCGCTGGGTACGGGGTTGCTCTGCCGGAGGCTGCCGCAACTTCCCAGGTGGGAGATGCTCTTGATGGGGGGAGGGTCTAAGCCGAAAAAGTTCCAGGCAGAAGAAGCCTAACTAGTGCTTATTAAGTCTCTCTGTTCCAGACGTCCACTATCTTATTAAACCTTCCCTGTTTTACTGAGAAGGAAACCACCATGCTGAGAAGTTTGCAATAGGGAGCTGGTAGCAACTTTGAAGCAGGAAACTGTGGCCCAAATCATTAGAGAAATGCCTGAATCGTGTTTTCTGTTGATATTTCCTAAGCACCTAGATGTAGGGAATAGAAATAAATGGCTCCCTCTGTCTCATCCCCTTCCTGCCCTCTGAGAGGCAGCTGTGAATGCGTGCTTCCTTTCTGGGGGTGCAGATACTTTCTGGACCAACCCTCAGTACCGTCTGAAGCTCCTGGAGGAGGACGATGACCCTGATGACTCGGAGGTGATTTGCAGCTTCCTGGTGGCCCTGATGCAGAAGAACCGGCGGAAGGACCGGAAGCTAGGGGCCAGTCTCTTCACCATTGGCTTCGCCATCTACGAG-3’

Minigene-E10I10E11-MUT

5’-

GATGTCCTATGAGGATTTCATCTACCATTTCACAAAGTTGGAGATCTGCAACCTCACGGCCGATGCTCTGCAGTCTGACAAGCTTCAGACCTGGACAGTGTCTGTGAACGAGGGCCGCTGGGTACGGGGTTGCTCTGCCGGAGGCTGCCGCAACTTCCCAGGTGGAAGATGCTCTTGATGGGGGGAGGGTCTAAGCCGAAAAAGTTCCAGGCAGAAGAAGCCTAACTAGTGCTTATTAAGTCTCTCTGTTCCAGACGTCCACTATCTTATTAAACCTTCCCTGTTTTACTGAGAAGGAAACCACCATGCTGAGAAGTTTGCAATAGGGAGCTGGTAGCAACTTTGAAGCAGGAAACTGTGGCCCAAATCATTAGAGAAATGCCTGAATCGTGTTTTCTGTTGATATTTCCTAAGCACCTAGATGTAGGGAATAGAAATAAATGGCTCCCTCTGTCTCATCCCCTTCCTGCCCTCTGAGAGGCAGCTGTGAATGCGTGCTTCCTTTCTGGGGGTGCAGATACTTTCTGGACCAACCCTCAGTACCGTCTGAAGCTCCTGGAGGAGGACGATGACCCTGATGACTCGGAGGTGATTTGCAGCTTCCTGGTGGCCCTGATGCAGAAGAACCGGCGGAAGGACCGGAAGCTAGGGGCCAGTCTCTTCACCATTGGCTTCGCCATCTACGAG-3’

primer sequences used for site-directed mutagenesis

+30G>A-F

CCCTTACCtTTCTGGGTCTTCCTGGGTTCTGG

+30G>A-R

ACCCAGAAaGGTAAGGGTGGGGAAGAGAGGGG

+5G>A-F

CAGGTGGaAGATGCTCTTGATGGGGGGAGGGT

+5G>A-R

AGAGCATCTtCCACCTGGGAAGTTGCGGCAGC
