# Supplementary material for: Correction of aberrant splicing caused by intronic CAPN3 pathogenic variants using RNA-targeted therapeutic strategies in limb-girdle muscular dystrophy type R1
Source: Orphanet J Rare Dis. 2026 Jun 13;21:292. doi: 10.1186/s13023-026-04451-x (PMC13495512; doi:10.1186/s13023-026-04451-x)
Supplement: Supplementary file 5 [file 13023_2026_4451_MOESM3_ESM.docx]

All sequence variants in this manuscript have been verified using Mutalyzer.
